# Using AuNPs-DNA Walker with Fluorophores Detects the Hepatitis Virus Rapidly

**DOI:** 10.3390/bios14080370

**Published:** 2024-07-29

**Authors:** Baining Sun, Chenxiang Zheng, Dun Pan, Leer Shen, Wan Zhang, Xiaohua Chen, Yanqin Wen, Yongyong Shi

**Affiliations:** 1 Bio-X Institutes, Key Laboratory for the Genetics of Developmental and Neuropsychiatric Disorders (Ministry of Education), Shanghai Jiao Tong University, Shanghai 200030, China; hibatsuna1827@sjtu.edu.cn (B.S.); cxzheng@sjtu.edu.cn (C.Z.); pandun@sjtu.edu.cn (D.P.); wanzh318@sjtu.edu.cn (W.Z.); shiyongyong@sjtu.edu.cn (Y.S.); 2 Department of Infectious Diseases, Shanghai Sixth People’s Hospital Affiliated to Shanghai Jiao Tong University School of Medicine, Shanghai 200233, China; rachel_slr@sjtu.edu.cn

**Keywords:** hepatitis viruses, gold nanoparticles, DNA walker

## Abstract

Viral hepatitis is a systemic infectious diseases caused by various hepatitis viruses, primarily leading to liver damage. It is widely prevalent worldwide, with hepatitis viruses categorized into five types: hepatitis A, B, C, D, and E, based on their etiology. Currently, the detection of hepatitis viruses relies on methods such as enzyme-linked immunosorbent assay (ELISA), immunoelectron microscopy to observe and identify viral particles, and in situ hybridization to detect viral DNA in tissues. However, these methods have limitations, including low sensitivity, high error rates in results, and potential false negative reactions due to occult serum infection conditions. To address these challenges, we have designed an AuNPs-DNA walker method that uses gold nanoparticles (AuNPs) and complementary DNA strands for detecting viral DNA fragments through a colorimetric assay and fluorescence detection. The DNA walker, attached to gold nanoparticles, comprises a long walking strand with a probe sequence bound and stem-loop structural strands featuring a modified fluorescent molecule at the 3′ end, which contains the DNAzyme structural domain. Upon the addition of virus fragments, the target sequence binds to the probe chains. Subsequently, the long walking strand is released and continuously hybridizes with the stem-loop structural strand. The DNAzyme undergoes hydrolytical cleavage by Mg^2+^, breaking the stem-loop structural strand into linear single strands. As a result of these structural changes, the negative charge density in the solution decreases, weakening spatial repulsion and rapidly reducing the stability of the DNA walker. This leads to aggregation upon the addition of a high-salt solution, accompanied by a color change. Virus typing can be performed through fluorescence detection. The innovative method can detect DNA/RNA fragments with high specificity for the target sequence, reaching concentrations as low as 1 nM. Overall, our approach offers a more convenient and reliable method for the detection of hepatitis viruses.

## 1. Introduction

Viral hepatitis is widely spread worldwide, and there are five types of hepatitis viruses classified according to etiology [1,2], namely hepatitis A, B, C, D, and E. China is a high incidence area of viral hepatitis. Hepatitis A was reported to exist in China for over 5000 years [3]. The population prevalence of hepatitis A (anti-HAV positive) is about 80%. There were more than 292 million HBsAg carriers worldwide in 2016 [4], including 120 million in China, while the number increased to 296 million in 2019 and 820,000 deaths worldwide [5]. About 1% of the world’s population is infected with HCV, and in some regions, such as West Africa or Central Africa, hepatitis C infections account for about 5% to 8% of the infected population [6]. The clinical manifestations of all viral hepatitis are similar, including mainly fatigue, loss of appetite, oil aversion, abnormal liver function, and jaundice in some cases [7]. In severe cases, acute liver failure and hepatitis cirrhosis may occur, as well as multiple complications such as hepatic encephalopathy, upper gastrointestinal bleeding, and hepatorenal syndrome, and some patients may even need liver transplantation to keep them alive later. About 887,000 people die every year from hepatitis B and related diseases, mainly related to advanced liver fibrosis and cirrhosis [4,5,8].

The hepatitis virus was identified in infected people and led to the development of diagnostic tests, molecular characterization, and propagation in cell culture [9]. Currently, the method to detect and diagnose hepatitis viruses relies on enzyme-linked immunosorbent assay (ELISA), radioimmunoassay, immunoelectron microscopy to observe and identify viral particles, and in situ hybridization to detect viral DNA in tissues [2,6,9,10,11,12,13]. Although enzyme-linked immunosorbent assay (ELISA) is relatively convenient, the detection sensitivity of this method is low and causes considerable errors. It may also lead to false negative results and missing detection due to the latent serum infection. Compared with serology, which relies on the concentration of the protein immune response, developed nucleic acid molecular detection technology is considered a more sensitive and accurate diagnostic method.

To detect diseases more quickly and accurately (such as the influenza virus genome and cancer markers), some new detection technologies are developed, such as DNA nanotechnology [14,15], the CRISPR/Cas system [16], electrochemical biosensors [17], etc. Nucleic acid detection has been widely used to detect hepatitis virus in samples of different origins, like blood, saliva, and other clinical specimens [7]. Hepatitis virus has been detected by techniques such as restriction fragment length polymorphism [18], Southern blotting [19], amplification based on nucleic acid sequencing [20], reverse transcription-PCR (RT-PCR) [21], antigen capture RT-PCR [22], etc. There are also many ways to test for viral load, such as ultraviolet (UV) spectrophotometry, polymerase chain reaction (PCR), real-time PCR (rt-PCR), digital PCR, loop-mediated isothermal amplification (LAMP), transcription-mediated amplification (TMA), nucleic acid sequence-based amplification (NASBA), rolling circle amplification (RCA) as well as electrochemical, quartz crystal microbalance, microcantilever, and surface plasmon resonance biosensors [4,5,23,24,25]. However, these methods have certain limitations. For example, PCR is not fit for short-length oligonucleotides; the cost of fluorescence assay analysis can be high as expensive reagents and instruments may be required. Therefore, developing convenient and sensitive virus DNA/RNA detection methods is urgently needed.

The gold nanoparticles (AuNPs) method is one of the most studied nanomaterials for biomedical applications. AuNPs can be functionalized with various biomolecules, such as nucleic acids or antibodies, to recognize and bind to specific targets [26]. AuNPs have been tested with impressive results as a biosensor, contrast agent, and therapeutic agent [27]. It has been reported that small-size AuNPs (about 3 nm) exhibit antitumor activity against breast cancer (MCF-7) cell lines and colon carcinoma (HCT-116) cell lines, but no cytotoxicity to the human embryonic normal kidney cell line (HEK 293) [28]. In addition, AuNPs can be connected to anti-cancer drugs through surface functionalization, thus playing a role in drug delivery. Small-size AuNPs were used to pair with the anti-cancer drug methotrexate (MTX) and evaluate the stability and specificity of its drug delivery [29]. In addition to drugs and proteins, DNA can also be linked to AuNP by forming covalent bonds with functional groups (such as amine or mercaptan groups) on the DNA molecule, or by hybridizing single-stranded DNA to complementary sequences attached to the AuNP surface [30]. AuNPs-based biosensors are mainly used to detect small molecules, DNA and proteins, and use AuNPs’ surface plasmonic resonance (SPR) characteristics to detect target molecules with high sensitivity and selectivity [31]. DNA nanotechnology has made great strides in the past year. The DNA walker mimics natural molecular motors by biasing chemical energy against Brownian motion [32,33,34]. The DNA walker can automatically move in one direction along the track without intervention. According to this characteristic, the synthetic DNA walker has been widely used in nucleic acid amplification detection in recent years [35,36,37,38].

In this work, we designed a DNA walker using gold nanoparticles (AuNPs) and DNA complementary strands to detect hepatitis viral DNA fragments using colorimetric assay and fluorescence detection. Our study shows that the DNA walker operates at room temperature without the requirement of protein enzymes and temperature controllers after adding the target sequences and obtains the color change of the solution and fluorescence intensity change as an output signal. The method detects DNA fragments down to 1 nM with high specificity for the target sequence. This method provides a more convenient method for the reliable detection of hepatitis viruses.

## 2. Materials and Methods

### 2.1. Apparatus

UV-vis absorption spectra and fluorescence emission spectra were measured with a UV Spectrometer (Thermo Fisher Technology (China) Co., Ltd., Shanghai, China) and Steady State and Transient State Fluorescence Spectrometer (Edinburgh Instruments, Livingston, Scotland, UK), respectively, at the Instrumental Analysis Center of Shanghai Jiao Tong University. The centrifuge was SORVALL Legend MICRO 17R (Thermo Fisher Technology (China) Co., Ltd., Shanghai, China).

### 2.2. Materials and Reagents

Gold nanoparticles were purchased from Xi’an ruixi Biological Technology Co., Ltd. (Xi’an, China) (20 nm in diameter and spherical). These AuNPs are 20 nm in diameter, spherical, and coated with sodium citrate. The color of the AuNPs colloid is red, and the UV-vis shows a maximum absorbance at 520 nm and owns a uniform size distribution with an average size of 20 nm (Figure S1). Tris (2-carboxyethyl) phosphine hydro-chloride (TCEP) was purchased from Sigma (Shanghai, China). Tris-base was purchased from Sangon Biotech Co., Ltd. (Shanghai, China). Boronic acid and ethylenediaminetetraacetic acid tetrasodium (EDTA) were supplied by Sinopharm Chemical Reagent Co., Ltd. (Shanghai, China). DNA sequences (Table S1) were synthesized and purified by Sangon Biotech Co., Ltd. (Shanghai, China).

### 2.3. Preparation of DNA Walker

The DNA walker consists of three different types of ssDNA: a long walking strand, two probe strands, and a stem–loop strand. The 5′ ends of the long walking chain and the stem–loop chain are modified with sulfhydryl groups so that they can be attached to the AuNPs’ surface. The 3′ end of the stem–loop chain is modified with fluorophores (FAM, ROX, and Cy5 were used for HAV, HBV, and HCV, respectively). The target sequence of the synthesized virus was the conserved region sequence, which was derived from NCBI (Table S1).

### 2.4. Preparation of AuNPs-DNA Walker

Long walking strands and blocking strands were mixed in one tube with a molar ratio of 1:3 in annealing buffer. The reaction solution was heated to 95 °C for 10 min and gradually cooled to room temperature to ensure that the blocking strands could completely bind with the walking strands. The stem–loop strands were also annealed from 95 °C to room temperature to form secondary structures. The above two groups of solutions were incubated with TCEP at a 1:50 molar ratio for 2 h at room temperature to reduce the formation of disulfide bonds. The AuNPs, locked walking strands, and stem–loop strands were mixed at a 1:20:200 molar ratio and incubated in darkness at 4 °C for 16 h. Then, the sodium chloride solution was gradually added to the above mixture at 40 min intervals to achieve a final concentration of 0.2 M of NaCl. The solution was incubated further in the dark at 4 °C for 24 h. After that, the solution was centrifuged at 4 °C at 13,000 rpm for 30 min to separate the AuNPs-DNA walker from the effluents. The AuNPs-DNA walker was washed three times with washing buffer and re-suspended in a reaction buffer as a working solution.

### 2.5. Colorimetric Detection and Fluorescence Detection of Virul Fragments

First, 3 μL of 10 μM of the target fragments was added to 160 μL of working solution containing 1 nM of the AuNPs-DNA walker and performed at room temperature for 3 h. Then, the above solution was centrifuged at 13,000 rpm (16,600 rcf) for 30 min at 4 °C, and 110 μL of the supernatant was measured for fluorescence study. Take HAV, for example: the AuNPs-DNA walker for detecting HAV target sequences is labeled with FAM, whose excitation light is 490 nm and emission light is 520 nm. Fluorescence spectra were detected at an excitation light of 490 nm and emission light of 520 nm. The remaining 50 μL of solution was re-suspended, and 600 mM of MgCl_2_ was added, followed by visual observation and UV-vis absorption measurements.

## 3. Results

### 3.1. Operating Principle of the AuNPs-DNA Walker

**Figure 1:**
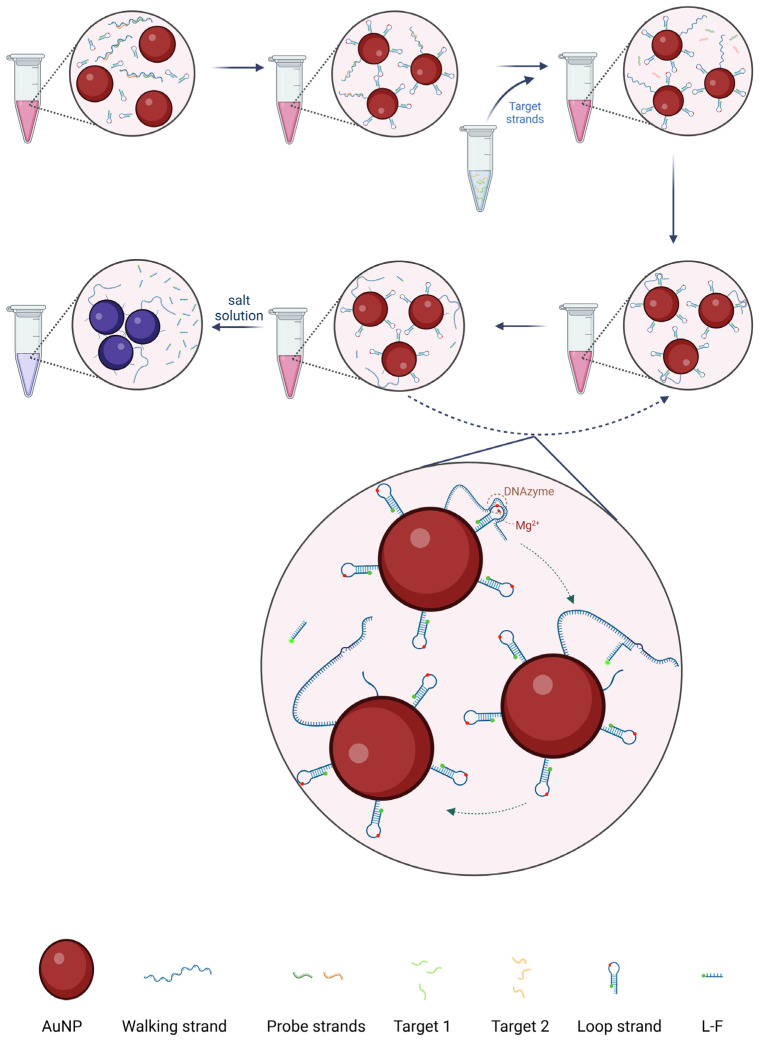
Schematic diagram of AuNPs-DNA walker for detecting viral DNA/RNA. AuNPs solution is pink. The color of the solution did not change after ssDNA coupling. After adding the target sequence, the color of the solution changes from pink to blue or bluish-purple visually after DNA strand replacement and salt addition.

We designed a DNA walker using gold nanoparticles (AuNPs) and DNA complementary strands to detect viral DNA fragments using a colorimetric assay and fluorescence detection (Figure 1). Attached to an AuNP, the DNA walker consists of a long walking strand with two probe strands bound to it and stem–loop structural strands with a modified fluorescent molecule at the 3′ end, which contains the DNAzyme structural domain. These DNAs were adsorbed on the AuNP by the sulfhydryl group at the 5′ end. Due to the repulsion between DNA, the electrostatic field force between the AuNPs became more extensive, so the aggregation phenomenon did not occur. The color of the AuNPs-DNA walker working solution was still pink. After the virus fragment was added, the target sequences would be wholly bound to the probe. The long walking chain would be released and continuously hybridized with the stem–loop structural chain. The DNAzyme would be hydrolytically cleaved by Mg^2+^, cutting the stem–loop structural chain into two linear single strands. One of the linear single chains was still attached to the AuNP, while the other containing the fluorescent molecule was released into the solution. When stem–loop structure chains were bound to the surface of the AuNP, fluorescence molecules were quenching due to the close distance between them. Then, the long walking chain would be released again and automatically found the next stem–loop chain. The previous step would be repeated until the DNAzyme hydrolyzed all the stem–loop chains on the AuNP into two short linear chains. After the step of DNAzyme cleaving, the part of the single chain containing fluorophores was released into the solution, and the quenching effect then disappeared. Changes in the DNA of AuNPs led to weaker repulsion between them, resulting in the aggregation of AuNPs when added to the salt solution. The color of the solution would change from pink to blue or bluish-purple. The colloidal stability before and after assembling the DNA walker was determined by the transmission electron microscopy (TEM) image and dynamic light scattering characterization (Figure S2). It can be seen that the AuNP before and after the function has a good dispersion, no large area aggregation phenomenon, and the AuNPs’ size increases.

### 3.2. Colorimetric Response of the AuNPs-DNA Walker to HAV Target Sequences

**Figure 2:**
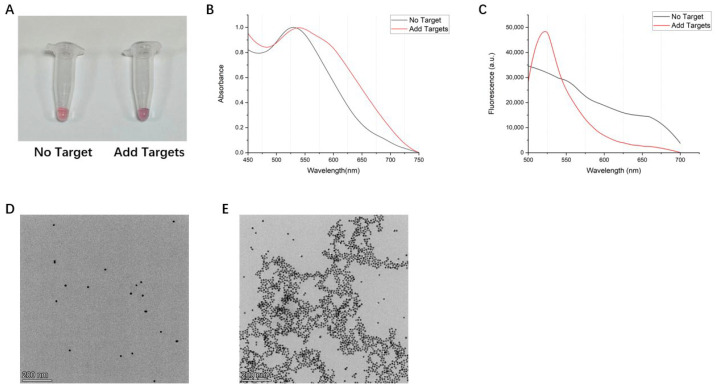
(**A**) The visual observation of AuNPs-DNA walker treated with/without target sequences. (**B**) UV–vis absorption spectra and (**C**) fluorescence spectra of the AuNPs-DNA walker in the absence or the presence of target sequences. Transmission electron microscopy (TEM) image of AuNPs-DNA walker treated with (**D**)/without (**E**) target sequences.

**Figure 3:**
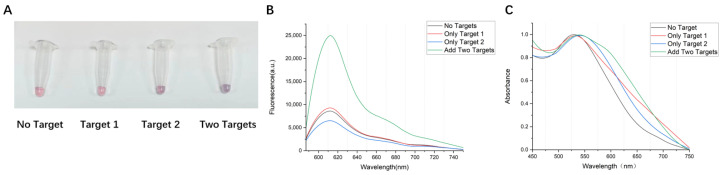

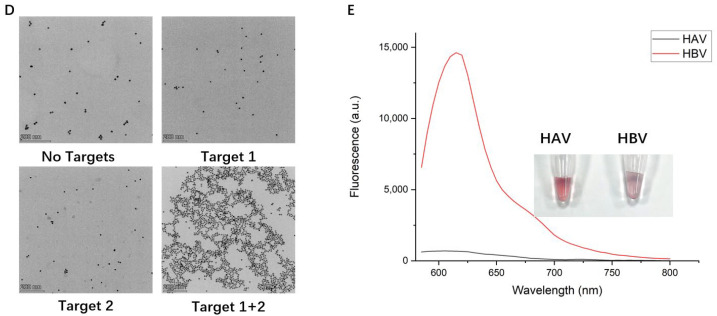
(**A**) Visual observation, (**B**) fluorescence spectra, (**C**) UV–vis absorption spectra, and TEM images (**D**) of AuNPs-DNA walker treated with no target, only one target, and both targets. (**E**) Fluorescence spectra and visual differentiation from HAV target sequences and HBV target sequences.

To test whether the AuNPs-DNA walker functioned as expected, we added the HAV target sequence to the AuNPs-DNA walker working fluid. Then, we observed the color change of the solution, UV-vis detection, and fluorescence spectrum detection of the supernatant. As a result, as shown in Figure 2A, after we added the HAV target sequence and MgCl_2_ solution, the color changed from pink to bluish-purple compared to the solution without the virus target sequence. UV-vis showed a red shift in the maximum absorbance from 520 nm to 550 nm, and a new absorption band appeared at 610 nm (Figure 2B). As a blank control, the color of the ordinary AuNPs solution without ssDNA was red, and UV-vis showed a peak at 520 nm (Figure S1). The results of the fluorescence spectrum detection showed that the AuNPs-DNA walker worked after adding the target sequence. The solution contained a linear single chain containing fluorophore FAM released after the DNAzyme hydrolyzed the stem–loop chain, and the emission light peak appeared at 519 nm (Figure 2C). The transmission electron microscopy (TEM) image showed the dispersion of the AuNPs-DNA walker with or without the addition of target sequences (Figure 2D,E). With this method, we also successfully detected the inclusion of HBV (Figure 3) and HCV target sequences (Figure S3): the visual observation and UV–vis absorption spectra of normal AuNPs.

To determine the optimal NaCl concentration to promote DNA binding to AuNPs, we developed an NaCl concentration gradient experiment and screened it using UV-vis detection and fluorescence spectroscopy. After mixing ssDNA with AuNPs, a certain amount of 2 M of NaCl solution was added to make the final concentrations of 0.05 M, 0.1 M, 0.2 M, 0.3 M, and 0.4 M, respectively. We explored the color changes of adding 600 mM of MgCl_2_ solution into the AuNPs-DNA walker with different final concentrations of NaCl. The color turns purple at 0.05 M and 0.1 M, there is no significant change in color at 0.2 M, and the color becomes lighter at 0.3 M and 0.4 M (Figure S4A). In addition, we added 1.5 μL of 10 μM of the target sequence chains into the AuNPs-DNA walker with different final concentrations of NaCl, and performed at room temperature for 3 h, centrifuged at 13,000 rpm, and the supernatant was taken to detect the fluorescence spectrum. The results show that the fluorescence intensity was the highest at 0.2 M concentration, which meant that the binding amount of ssDNA and AuNPs was the highest at this concentration (Figure S4B).

### 3.3. Specific Detection of the AuNPs-DNA Walker

To detect the specificity of the AuNPs-DNA walker, we added target sequence 1, target sequence 2, and both target sequences to the AuNPs-DNA walker working solution, respectively. After the salt solution was added, the color of the solution without the target sequence and the solution with target sequence 1 did not change significantly. In contrast, the color of the solution with target sequence 2 changed slightly, and the color of the solution with both target sequences turned bluish-purple (Figure 3A). The fluorescence spectra showed a noticeable intensity difference between the four tubes (Figure 3B), while UV-vis detection was consistent with the color change of the solution (Figure 3C). Although there were color changes and red shifts in the tube added to target sequence 2, there was no apparent fluorescent luminescence. This might be related to the location of the long walking chain exposed after the target sequence binds to the probe chain. TEM showed the dispersion of the AuNPs-DNA walker with or without the addition of target sequences (Figure 3D). We also used the HAV target sequences as a confusion chain and added them into the HBV-specific AuNPs-DNA walker solution. The results show that the AuNPs-DNA walker could distinguish the non-target sequence from the target sequence with reasonable specificity (Figure 3E).

### 3.4. Test of the AuNPs-DNA Walker to Virus Target Fragments

**Figure 4:**
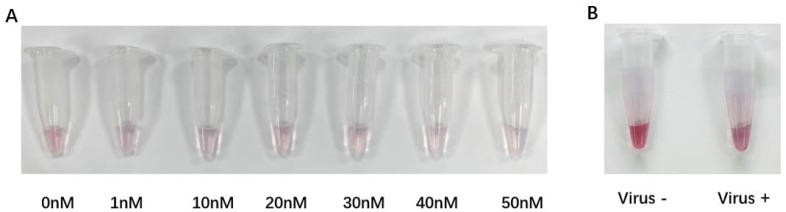
(**A**) Visual observation of the AuNPs-DNA walker treated with different concentrations of target chains within the range from 0 to 50 nM. (**B**) Visual observation of the AuNPs-DNA walker treated with/without HCV virus fragments.

Firstly, we examined the minimum detection concentration of the target sequence by this method. We designed a concentration gradient from 0 nM to 50 nM for the HCV target sequence and tested it with the AuNPs-DNA walker. The results show that as the concentration of the double target sequences increased, the color of the detection solution changed from red to purple at 1 nM (Figure 4A). The low concentration mixture was further detected by transmission electron microscopy. It was found that AuNPs only partially coagulated at a low concentration, which was consistent with the change in color of the solution under visual observation, which was not as obvious as that under a high concentration (Figure S5). To verify the success of this method on the viral fragments extracted from the blood samples of infected patients, we collected blood samples from HCV patients. We obtained HCV single-stranded DNA samples by RT-PCR and asymmetric amplification. HCV target fragments were added into the solution containing the AuNPs-DNA walker, shaken for 30 min, and then performed at room temperature for 2 h, followed by salt addition and visual observation. The concentration of the HCV sample used was 200 copies/mL, and the results show that a significant color change in the solution could be seen visually (Figure 4B). The detection limit was estimated to be 200 copies/mL, which could meet the requirements of individuals with a high diagnostic transmission rate and short duration of symptom onset.

## 4. Discussion

In this study, we combined gold nanoparticles and DNA strand replacement technology to design an AuNPs-DNA walker for the rapid detection of hepatitis virus. We took conserved region sequences of different types of hepatitis viruses as target sequences, connected corresponding different fluorophores at the 3′ end of stem-loop chain (HAV for FAM, HBV for ROX, HCV for Cy5), and used fluorescence spectrum detection technology to distinguish hepatitis virus types. The method does not require the amplification/reverse transcription of viral DNA/RNA in the blood samples of hepatitis patients, and the minimum detection threshold is set to 200 copies/mL.

The electrochemical method was proposed for the detection of the hepatitis C virus (HCV) RNA level and identification of the HCV-1b genotype based on the site-specific cleavage of BamHI endonuclease combined with gold nanoparticles (AuNPs) signal amplification [39]. This method’s procedures include reverse transcription, PCR amplification, and electrochemical detection. In 2014, it was reported that a new assay using magnetic nanoparticles and unmodified cationic gold nanoparticles was developed for detecting hepatitis C virus in serum samples and tested in clinical samples [40]. The specificity and sensitivity were 96% and 96.5%, respectively, and the detection limit was 15 IU/mL. Meanwhile, in 2017, Sherif Shawky et al. used RT-PCR and nano-assay to quantitatively detect HCV RNA samples with detection limits as low as 4.57 IU/mL [41]. In response to the low abundance of viral nucleic acid in the blood of early patients, Clarke et al. obtained a high-quality, reproducible surface-to-enhanced raman spectroscopy (SERS) with report-modified gold nanoparticles to detect anti-HCV antibodies in the blood samples of HCV-infected patients [42]. Feng Tao et al. then combined the DNA walker and catalytic hairpin assembly (CHA) to perform the targeted detection of HBV DNA. Their method achieves a wide detection range of 0.5 nM to 50 nM, with detection limits as low as 0.20 nM [37]. An isothermal amplification technique based on digital ring street was developed in 2020, and the HCV viral nucleic acid in the blood samples was detected by silica coating and AuNPs [43]. This system could detect HBV-DNA at a concentration of 10 to 1 × 10^4^ copies/μL. During the COVID-19 pandemic, there have been many reports of viral RNA detection using colorimetric sensing methods of nanoparticles to detect whether patients are infected with COVID-19 quickly [36,44]. Jiafeng Pan et al. combined DNA logic gates with various fluorophores to identify COVID-19, SARS-CoV, and Bat-SL-CoVZC45 simultaneously [15]. However, this method needs to add exonuclease III to assist operation, which is inconvenient in practical application. To provide more accurate detection, Maha Alafeef et al. used a dual-targeted approach to detect early infected samples with low viral loads, reducing the detection limit to 10 copies/μL [45]. Kai Zhang et al. optimized DNA probes and nanomaterials, and lowered the detection limit to 59 aM based on the electrochemical luminescence detection method [46]. Recently, Laibao Zheng et al. utilized 3D-DNA walking nanomachines for the sensitive detection of hepatitis C virus. This method has shown excellent sensitivity in detecting HCV with a detection limit of 42.4 pM and a linear range of 100 pM to 2 nM [38].

Our method combines the DNA walker and AuNPs. After adding the target sequence, through DNA strand replacement and DNA walker operation, the mutual repulsion between AuNPs decreased, and the resistance to salt solution weakened, so that the color of the solution changed visually. This method is more practical for the preliminary screening of people in remote areas without instrument detection. In addition, our methods adopted AuNPs and single-strand DNA instead of the traditional ELISA test. Under the condition that the price is nearly the same, traditional ELISA kits can serve 48 people, whereas our AuNPs-DNA walker can accommodate over 200 people. In terms of time, traditional ELISA tests typically require 4–6 h, whereas ours only take 3 h and are much simpler to operate. Therefore, compared to traditional testing methods, our approach significantly enhances cost-effectiveness. Although our method can detect the type of hepatitis virus more easily, it cannot further detect its corresponding subtype, and for the double-DNA strand virus of HBV, it is still necessary to convert the viral DNA into ssDNA by asymmetric amplification technology before detection. The process can have conditions such as base mutations that can lead to false positives/false negatives. These are the limitations of the method, which can be further studied in the future.

## 5. Conclusions

In summary, we developed an AuNPs-DNA walker using gold nanoparticles (AuNPs) and DNA complementary strands for the detection of viral DNA fragments through colorimetric and fluorescence assays. Our AuNPs-DNA walker can detect viral target sequences with high specificity down to low concentrations (1 nM). Compared to commonly used methods, such as ELISA, our design offers several advantages. It eliminates the need for specialized equipment and does not require proteases. Additionally, it can simultaneously identify the presence of three viral genes. The AuNPs-DNA walker is straightforward to perform at room temperature, yielding test results quickly and producing clear, visualized outcomes. Furthermore, this method is cost-effective and easily scalable, making it accessible for widespread use. Overall, our approach provides a more convenient and reliable solution for the detection of hepatitis viruses.

## Data Availability

Data will be made available on request.

## Supplementary Materials

The following supporting information can be downloaded at: https://www.mdpi.com/article/10.3390/bios14080370/s1, Figure S1: The form and color of AuNPs; Figure S2: Size and stability before and after AuNP function. Figure S3: UV–vis absorption spectra and fluorescence spectra of the AuNPs-DNA Walker in the absence or the presence of HCV target DNA fragments. Figure S4: Color changes and fluorescence spectra of AuNPs-DNA walker aged with NaCl at different final concentrations after adding salt solution; Figure S5: TEM image of adding different concentration of target sequences. Table S1: Sequences of oligonucleotides used in this study.
