# Supplementary material for: Using AuNPs-DNA Walker with Fluorophores Detects the Hepatitis Virus Rapidly
Source: Biosensors (Basel). 2024 Jul 29;14(8):370. doi: 10.3390/bios14080370 (PMC11353006; doi:10.3390/bios14080370)
Supplement: Supplementary file 1 [file biosensors-14-00370-s001.zip › biosensors-3000222-supplementary.pdf]

Supplement Material

The buffer used in this study.
annealing buffer: 25 mM Tris-acetate, 200 mM NaCl, pH 8.0;
washing buffer: 25 mM Tris-acetate, pH 8.0;
storage buffer: 25 mM Tris-acetate, 100 mM NaCl, pH 8.0;
reaction buffer: 5 mM Tris-acetate, 225 mM NaCl, 20 mM MgCl2, pH 8.0.

Table S1. Sequences of oligonucleotides used in this study.

Name	Sequence (5′ to 3′)
HAV-Walking strand	HS-SH-(T)42GATTCAGTTGGTCAGC*TCTCTTC*TCCGAGCCGGTCGAAA*TAGT*GTTGGACATGATTCTG
HBV-Walking strand	HS-SH-(T)42CGCATCAGGACTCCTA*TCTCTTC*TCCGAGCCGGTCGAAA*TAGT*AGTTCAGGAACAGTGA
HCV-Walking strand	HS-SH-(T)42GCTTGCGAGTGCCCCG*TCTCTTC*TCCGAGCCGGTCGAAA*TAGT*GCTCAATGCCTGGAGA
HAV-Stem-loop strand	HS-SH-(T)6GGCTGT*ACACTAT*/rA/G*GAAGAGA*TTCAGCCCGCGTTTTTTTCGCG-FAM
HBV-Stem-loop strand	HS-SH-(T)6GGCTGT*CTACTAT*/rA/G*GAAGAGA*TTCAGCCCGCGTTTTTTTCGCG-ROX
HCV-Stem-loop strand	HS-SH-(T)6GGCTGT*GCACTAT*/rA/G*GAAGAGA*TTCAGCCCGCGTTTTTTTCGCG-Cy5
Target-A1	GTTGGACATGATTCTGGAGGTTT
Target-A2	TGCTTCAGATTCAGTTGGTCAGC
Target-B1	AGTTCAGGAACAGTGAGCCCTAC
Target-B2	AGAACATCGCATCAGGACTCCTA
Target-C1	GCTCAATGCCTGGAGATTTGGGC
Target-C2	ATAGGGTGCTTGCGAGTGCCCCG
Probe-A1	AAACCTCCAGAATCATGTCCAAC*ACTA*
Probe-A2	*AAGAGA*GCTGACCAACTGAATCTGAAGCA
Probe-B1	GTAGGGCTCACTGTTCCTGAACT*ACTA*
Probe-B2	*AAGAGA*TAGGAGTCCTGATGCGATGTTCT
Probe-C1	GCCCAAATCTCCAGGCATTGAGC*ACTA*
Probe-C2	*AAGAGA*CGGGGCACTCGCAAGCACCCTAT

The complementary bases between the target sequences and probes of HAV, HBV, and HCV are labeled with different shades of red, blue, and yellow. The catalytic core region of DNAzyme in Walking strands and stem-loop strands are marked in green, while its

flanking binding regions and the corresponding complementary bases are marked in purple italics.

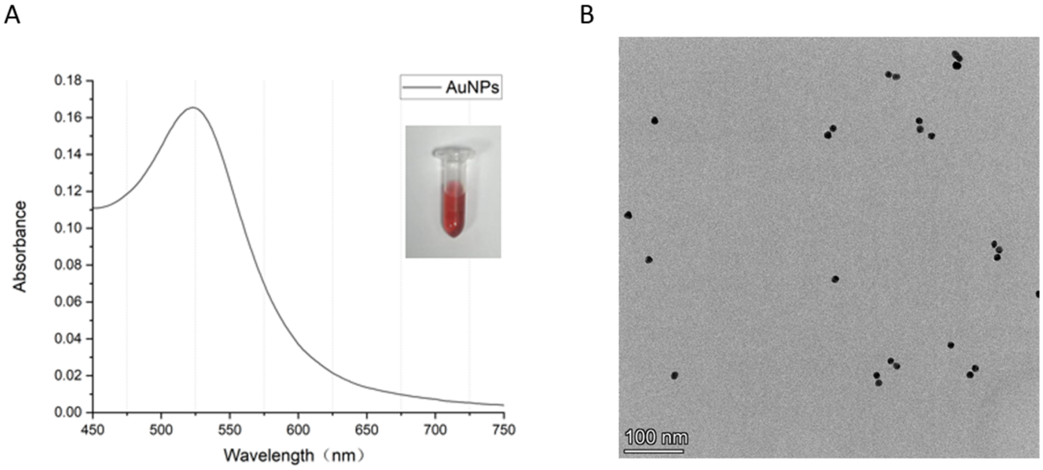

Figure S1. The form and color of AuNPs. The form and color of AuNPs. (A) The visual observation and UV–vis absorption spectra of AuNPs. (B) Transmission electron microscopy (TEM) image of AuNPs.

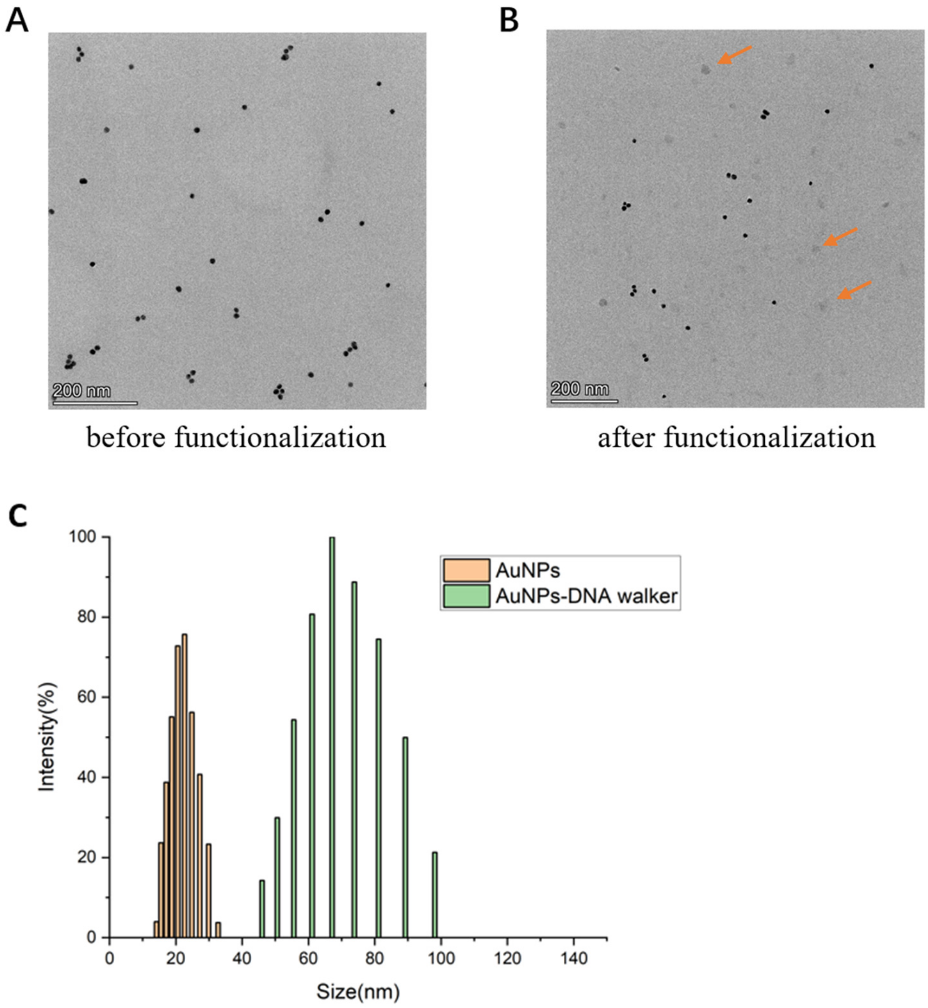

Figure S2. Size and stability before and after AuNP function. (A) is the TEM image before AuNP functionalization under transmission electron microscope, (B) is the TEM image after AuNP functionalization under transmission electron microscope, and the gray and black irregularities in the background are the salt crystals remaining after liquid evaporation. (C) Test the size comparison before and after AuNP function for DLS.

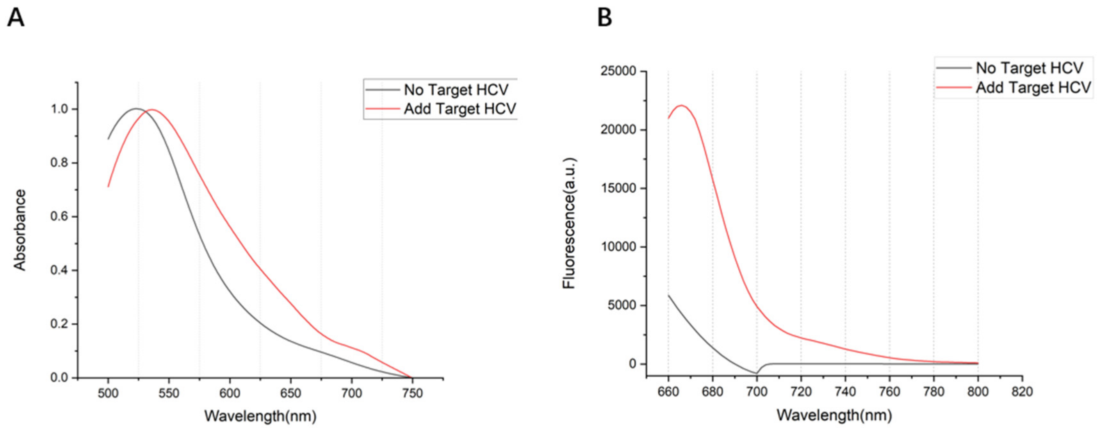

Figure S3. (A)UV–vis absorption spectra and (B) Fluorescence spectra of the AuNPs-DNA Walker in the absence or the presence of HCV target DNA fragments.

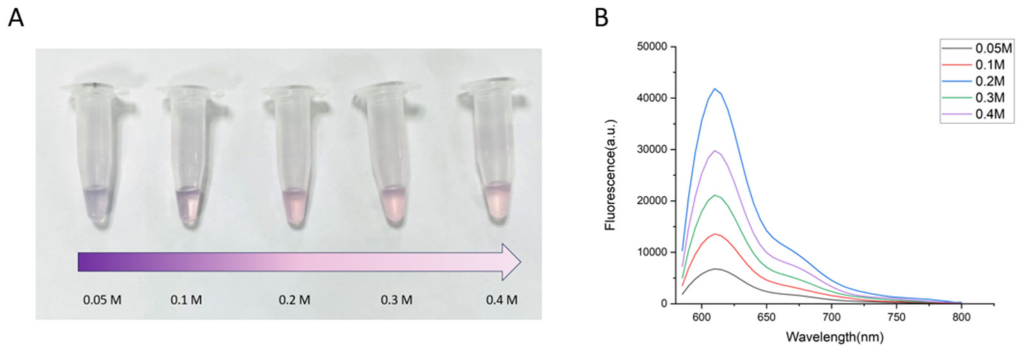

Figure S4. Color changes (A) and fluorescence spectra (B) of AuNPs-DNA walker aged with NaCl at different final concentrations after adding salt solution. The black is 0.05 M NaCl, the red is 0.1 M NaCl, the blue is 0.2 M NaCl, the green is 0.3 M NaCl, and the purple is 0.4 M NaCl.

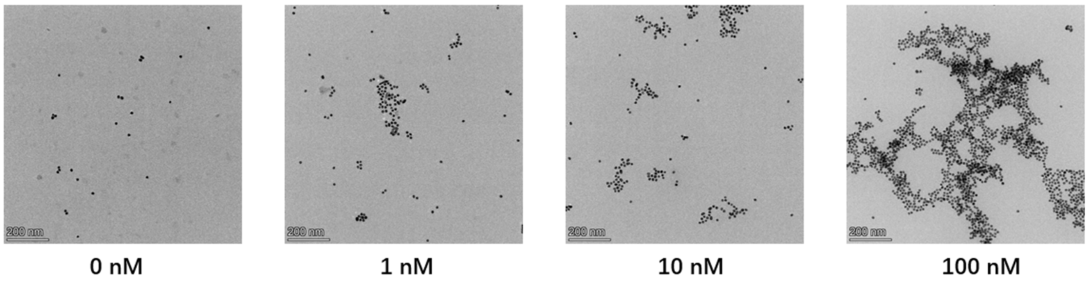

Figure S5. TEM image of adding different concentration of target sequences.
